# Comparative Pathogenesis of Two Lineages of Powassan Virus Reveals Distinct Clinical Outcome, Neuropathology, and Inflammation

**DOI:** 10.3390/v16060820

**Published:** 2024-05-22

**Authors:** Erin S. Reynolds, Charles E. Hart, Jacob T. Nelson, Brandon J. Marzullo, Allen T. Esterly, Dakota N. Paine, Jessica Crooker, Paul T. Massa, Saravanan Thangamani

**Affiliations:** 1 Department of Microbiology and Immunology, SUNY Upstate Medical University, Syracuse, NY 13210, USAallen.esterly@yale.edu (A.T.E.);; 2 SUNY Center for Vector-Borne Diseases, SUNY Upstate Medical University, Syracuse, NY 13210, USA; 3 Institute for Global Health and Translational Science, SUNY Upstate Medical University, Syracuse, NY 13210, USA; 4 Department of Biochemistry, Jacobs School of Medicine and Biomedical Sciences, SUNY Buffalo, Buffalo, NY 14203, USA; 5 Genomics and Bioinformatics Core, New York State Center of Excellence Bioinformatics & Life Sciences, SUNY Buffalo, Buffalo, NY 14203, USA; 6 Department of Neurology, SUNY Upstate Medical University, Syracuse, NY 13210, USA

**Keywords:** Powassan virus, deer tick virus, Neuropathogenesis, tick-borne flavivirus

## Abstract

**Author Summary:**

The Powassan virus causes a nationally notifiable disease which can cause severe neurological disease in humans owing to the lack of approved vaccines or therapeutics. Although two distinct lineages circulate in North America, clinical differentiation does not typically occur because pathology has been assumed to be similar between lineages. In this work, a direct comparison of lineage I (Powassan virus) and lineage II (deer tick virus) demonstrated distinct differences in the clinical presentation, pathology of the central nervous system, and immune response in immunocompetent mice. These differences suggest that the deer tick virus and Powassan virus do not utilize the same mechanisms for neuroinvasion and dissemination within the CNS. This is clinically relevant as the development of treatments and therapeutics needs to be evaluated for these virus lineages.

**Abstract:**

Tick-borne flaviviruses (TBFV) can cause severe neuroinvasive disease which may result in death or long-term neurological deficit in over 50% of survivors. Multiple mechanisms for invasion of the central nervous system (CNS) by flaviviruses have been proposed including axonal transport, transcytosis, endothelial infection, and Trojan horse routes. Flaviviruses may utilize different or multiple mechanisms of neuroinvasion depending on the specific virus, infection site, and host variability. In this work we have shown that the infection of BALB/cJ mice with either Powassan virus lineage I (Powassan virus) or lineage II (deer tick virus) results in distinct spatial tropism of infection in the CNS which correlates with unique clinical presentations for each lineage. Comparative transcriptomics of infected brains demonstrates the activation of different immune pathways and downstream host responses. Ultimately, the comparative pathology and transcriptomics are congruent with different clinical signs in a murine model. These results suggest that the different disease presentations occur in clinical cases due to the inherent differences in the two lineages of Powassan virus.

## 1. Introduction

The Powassan virus is the only known TBFV circulating in North America and has two distinct genetic lineages. The prototypical Powassan virus, lineage I (POWV), was isolated in 1958 and known to be vectored by *Ixodes cookei* ticks [1,2]. Lineage II Powassan virus, also known as deer tick virus (DTV), was initially isolated in 1995 and is vectored primarily by *Ixodes scapularis* ticks [3,4]. The Powassan virus and DTV share approximately 84% of their nucleotide sequence identity and 94% of their amino acid identity but are serologically indistinguishable [5,6,7,8]. The number of cases and geographic range for both the POWV and DTV have increased, coinciding with the expansion of *Ixodes* ticks and increased surveillance. In addition, recent studies have demonstrated that Powassan lineages may not only be vectored by *Ixodes* ticks but may also be transmitted by some species of *Dermacentor*, *Amblyomma*, and *Haemaphysalis* ticks [9,10,11]. 

Infection with the POWV is usually asymptomatic; however, some individuals will experience a febrile illness that can progress to severe neuroinvasive disease that may present with meningoencephalitis or paralysis. Case fatality rates are estimated to be between 10% and 15%, and over 50% of POWV survivors experience long-term neurological sequelae [5,12]. While there have been advances in the study of POWV neuroinvasive disease, the underlying pathology is not completely defined, especially in relation to the similarly pathogenic DTV. The Powassan virus disease is a nationally notifiable condition, but the differentiation of lineages is not required [13]. When lineage differentiation is performed for human cases, it is most often performed through the sequencing of viral RNA as methods such as neutralization and immunohistochemistry use antibodies which are cross-reactive [14,15,16,17].

The neuropathology seen in the POWV is currently believed to be mediated by a combined activity of inflammatory cytokines and extravasating leukocytes in the CNS. Of possible relevance to differential POWV and DTV pathogenesis, infections by clinically important TBFV may provide important clues on what determines particular disease outcomes. For instance, the levels of TNFα, IL-1β, IL-6, and IL-8 in the CNS have been associated with the proinflammatory breakdown of the BBB through the loss of tight junctions, with cytokine-induced MMP-9 being crucial for neuroinvasive disease with the tick-borne encephalitis virus (TBEV) [18,19]. Once the integrity of the BBB has been compromised, the neuronal destruction and pathology associated with TBEV infection are driven by the migration of CD8+ T-lymphocytes and NK cells [20]. Besides immune-mediated neuronal destruction, neuronal demise can occur throughout the CNS by direct virus-induced cell death, as seen in connected neuronal network in brains infected by the related Langat virus (LGTV) [21]. Noting the differences in CNS pathogenesis of the TBFV, we describe here the essential differences in the pathogenicity of the POWV and DTV which are of relevance, as historically, these viruses were assumed to be interchangeable, making these findings novel and clinically relevant. As such, we propose that the present studies support a reconsideration of distinct pathogenetic outcomes of infection by POWV lineages in humans.

## 2. Results

### 2.1. Immunocompetent Mice Inoculated with DTV and POWV Had Different Clinical Disease Presentations

**Figure 1:**
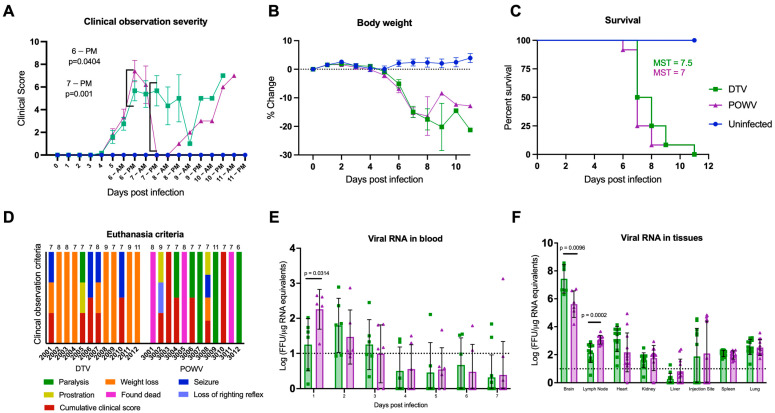
Clinical presentation of DTV and POWV infections. Mice (*n* = 12 per group) were inoculated via the footpad with 10^3^ FFU of media (blue circles), DTV (green squares), or POWV (purple triangles) and monitored for signs of disease for up to 11 dpi. (**A**) Clinical signs of disease were categorized and scored by severity (Table S1). Significant differences in DTV and POWV mice clinical scores occurred at the PM observations on 6 dpi and 7 dpi. (**B**) Percent weight change from pre-infection. Comparisons on (**A**,**B**) were made using one-way ANOVA with Šίdάk multiple comparisons test. Error bars indicate SEM. (**C**) Survival of mice across all treatments were evaluated using the Mantel–Cox log-rank test and median survival times (MST) were calculated. (**D**) Humane euthanasia was performed when mice met specific criteria. The cause(s) of mortality were summarized for each animal to demonstrate differences between DTV and POWV. Each colorized bar indicates the reason(s) for euthanasia. The numbers above the bars indicate the day of euthanasia post-infection. (**E**,**F**) Blood was collected from alternating cohorts of male (*n* = 3) and female (*n* = 3) mice from 0 to 7 dpi and at termination blood and tissues (brain, popliteal lymph node, heart, kidney, liver, injection site, spleen, and lung) were collected from all animals to determine viral loads via rt-qPCR. For brain analysis, six animals per group (3M and 3F) were tested and for all other tissues twelve animals (6M and 6F) were tested. Viral loads are expressed as Log FFU equivalents per microgram of RNA after normalization to a standard curve. Comparisons on (**D**,**E**) were performed using two-tailed Student’s *t*-test with Welch’s correction. For statistically significant comparisons, *p*-values are provided on the figure panels.

We investigated whether equivalent doses of either the DTV or POWV resulted in a different clinical presentation in six-week-old BALB/cJ mice. The first outward signs of illness occurred at 5 days post infection (dpi) when weight loss was observed in both infected groups (Figure 1A,B). Although weight loss occurred across both infected groups, it was more severe in the DTV-infected mice. Seventy-five percent of DTV-infected mice (*n* = 9) and 8% of POWV-infected mice (*n* = 1) reached a loss of 20% or greater from their baseline weight before reaching a cumulative clinical score requiring humane euthanasia (Figure 1D). Starting at 6 dpi, generalized signs of febrile illness occurred in both groups including a ruffled coat, hunched posture, lethargy, and rapid or labored respiration. Ocular discharge and closure of one or both eyes occurred in both the infected groups between 6 dpi and 8 dpi but was more common in the DTV-infected mice (50%, *n* = 6) than the POWV-infected mice (33%, *n* = 4). The onset of neurological illness occurred at 6 dpi in the POWV-infected mice and at 7 dpi for the DTV-infected mice and was markedly different between these groups. Forty-two percent of the DTV-infected mice presented with neurological illness, including seizure (*n* = 4) (Figure 1D) and a weak grip which progressed to paralysis (*n* = 1) (Figure 1D). In contrast, neurological illness was present in 83% of the POWV-infected mice, with 25% of the mice exhibiting multiple signs. The observed signs in the POWV-infected mice included a weak grip (*n* = 3), paresis (*n* = 3), loss of righting reflex/prostration (*n* = 2), seizure (*n* = 1), and paralysis (*n* = 5) (Figure 1D).

Mean clinical disease scores and percent weight change from baseline were compared between all groups until 8 dpi, after which sample sizes were too small for statistical comparison. Between 5 dpi and 8 dpi, there were statistically significant differences in the clinical disease scores between uninfected mice and each infected group and between DTV- and POWV-infected mice at the second daily observation on 6 dpi [*p* = 0.0404] and 7 dpi [*p* = 0.001] (Figure 1A). Body weight changes from baseline were significantly different between uninfected and each infected group starting at 6 dpi but not between DTV- and POWV-infected groups (Figure 1B).

Differences in mean clinical disease scores and percent weight change from baseline were also compared between male and female mice within a group. The DTV-infected male and female mice had significantly different clinical scores during the first daily observation at 7 dpi [*p* = 0.0298] (Figure S1A) and no significant differences were found in the POWV-infected male and female mice (Figure S1F). In addition, trends in weight loss were consistent between male and female DTV- and POWV-infected mice (Figure S1B,G). Overall, DTV-infected mice experienced a higher degree of weight loss than POWV-infected mice while POWV-infected mice experienced more loss of ambulation than DTV-infected mice.

### 2.2. Immunocompetent Mice Inoculated with DTV and POWV Had the Same Time to Death

Despite differences in clinical disease, all mice inoculated via footpad with 10^3^ FFU of DTV or POWV succumbed to disease between 6 and 11 dpi. Log-rank test (Mantel–Cox) did not show significant differences in survival between DTV- and POWV-infected mice (Figure 1C), having median survival times (MST) of 7.5 and 7 days, respectively. In addition, no significant differences were present between male and female mice within a group (Figure S1C,H) using the same analyses. The MST for DTV-infected mice was 7 days for males and 8.5 days for females, while the MST for POWV-infected mice was 7 days for both males and females.

### 2.3. Viral Titers Differ in Blood and Tissues of DTV- and POWV-Infected Mice

Viral RNA was detected in blood samples from all the DTV-infected mice and all except one POWV-infected mouse between 1 dpi and 3 dpi (Figure 1E). The viral RNA declined following 3 dpi and was not detected in blood samples after 9 dpi. The mean viremia for the DTV group peaked 2 dpi at 1.798 Log FFU/µg RNA equivalents, while the POWV group peaked 1 dpi at 2.258 Log FFU/µg RNA equivalents (Figure 1E). At 1 dpi, there was higher viremia in the POWV-infected mice than in the DTV-infected mice [*p* = 0.0314]. 

The detection of viral RNA in the organs demonstrated that the DTV and POWV disseminate throughout the body. Viral RNA was present in the brain, and popliteal lymph node from the injected footpad, spleen, and lung of all infected mice (Figure 1F). There was viral RNA detected in the heart and kidney of all the DTV-infected mice and 92% of the POWV-infected mice (*n* = 11). Viral RNA was present at the injection site for 50% of the mice in each infected group (*n* = 6). Limited viral RNA was found in the liver with either no viral RNA or RNA below the detection limit in 92% of the DTV-infected mice (*n* = 11) and 67% of the POWV-infected mice (*n* = 8).

The highest amounts of viral RNA were detected in the brains of both the infected groups, with an average of 7.43 Log FFU/µg RNA equivalents present in the DTV group and 5.62 Log FFU/µg RNA equivalents present in the POWV group (Figure 1F). The brains of the DTV-infected mice had higher viral loads than those of the POWV-infected mice [*p* = 0.0096], while the POWV-infected mice had higher viral loads in the lymph node [*p* = 0.0002]. A comparison of viral RNA present in the organs was also performed between the infected male and female mice, and the only significant difference was found in the livers of the POWV-infected mice (Figure S1I). 

### 2.4. Comparative Histopathology of Deer Tick Virus- and Powassan Virus-Infected Brains and Spinal Cords

**Figure 2:**
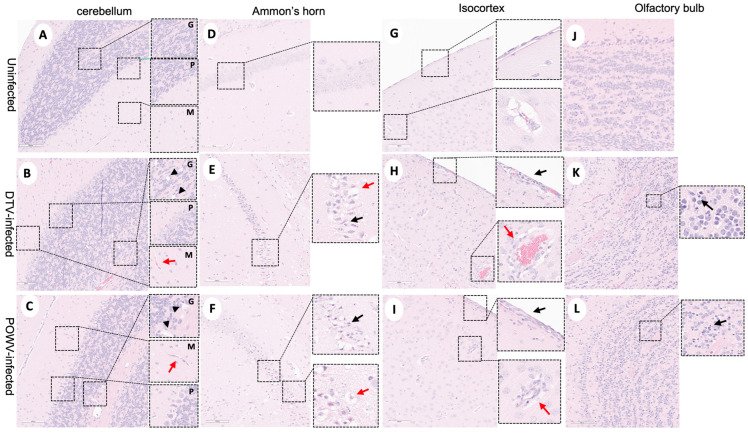
Neuropathological examination of DTV- and POWV-infected mouse brains. Brains were harvested at the time of euthanasia. Uninfected mouse with normal structures shown in (**A**) cerebellum with insets showing molecular cell layer—M, Purkinje cell layer—P, and granular cell layers—G, (**D**) Ammon’s horn of the hippocampus, (**G**) isocortex, and (**J**) olfactory bulb. Cerebellum of DTV- (**B**) and POWV- (**C**) infected mice with microgliosis (inset, red arrow) and neuronal cell necrosis (inset, black arrowheads). Ammon’s horn of the hippocampus from DTV- (**E**) and POWV- (**F**) infected mice with neuropil vacuolation (inset, red arrow) and hyper eosinophilic degenerating neurons (inset, black arrow). Isocortex of DTV (**H**) and POWV (**I**) with infiltration of leptomeninges (inset, black arrow) and perivascular cuffing (inset, red arrow). Olfactory bulb of DTV- (**K**) and POWV- (**L**) infected mice with neuronal cell necrosis in granular cell layers (black arrow).

**Figure 3:**
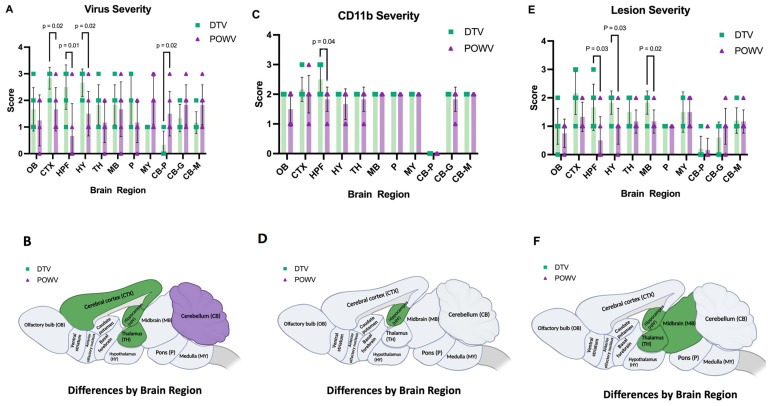
Severity of virus, CD11b, and lesions in DTV- and POWV-infected mouse brains by region. Brains were harvested at the time of euthanasia. (**A**) Virus severity in individual mice by region and (**B**) brain regions of DTV-infected mice had statistically more virus severity in CTX, HPF, and TH while POWV-infected mice had more virus severity in CB. (**C**) CD11b severity of individual mice by region. (**D**) Brain regions of DTV-infected mice had statistically significant increases in CD11b positive cells in HPF. (**E**) Severity of individual mice by region. (**F**) Brain regions of DTV-infected mice had statistically significant differences in severity in the HPF, MB, and TH regions. All comparisons performed using unpaired two-tailed Student’s *t*-test with Welch’s correction. For statistically significant comparisons, *p*-values are provided on the figure panels.

Histopathological changes were observed in the POWV- and DTV-infected mouse brains across multiple regions including the olfactory bulb (OB), cerebral cortex (CTX), hippocampal formation (HPF), hypothalamus (HY), thalamus (TH), midbrain (MB), pons (P), medulla (MY), and cerebellum (Purkinje layer: CB–P, granular layer: CB- G, molecular layer: CB–M) and scored by severity (Table S2). Infected mice exhibited microgliosis and neuronal necrosis in the cerebellum (Figure 2A–C) and olfactory bulb (Figure 2J–L), degenerating neurons and vacuolation of the neuropil were observed in Ammon’s horn of the hippocampal formation (Figure 2D–F), and the isocortex showed perivascular cuffing and meningoencephalitis (Figure 2G–I). The DTV-infected mice had higher lesion severity scores in the hippocampal formation, thalamus, and midbrain regions compared to the POWV-infected mice (Figure 3E,F).

**Figure 4:**
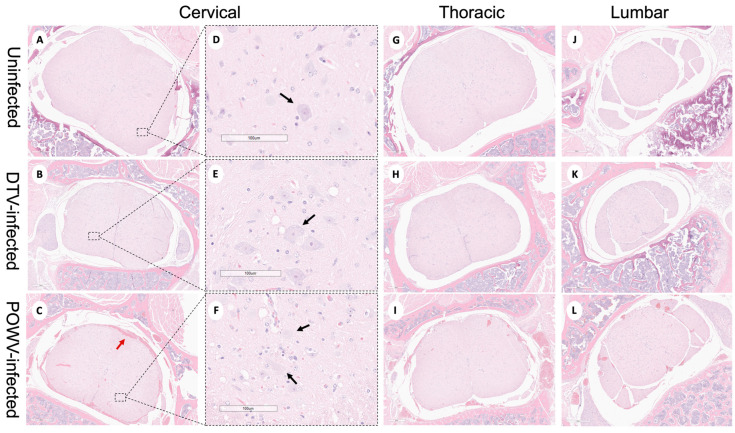
Neuropathological examination of DTV- and POWV-infected mouse spinal cords. Spinal cords were harvested at the time of euthanasia. Uninfected mouse with normal structures shown in (**A**,**D**) the cervical region (black arrow shows motor neuron in ventral horn), (**G**) thoracic region, and (**J**) lumbar region. DTV-infected mouse (**B**) cervical region, (**E**) motor neuron (black arrow) in dorsal horn, (**H**) thoracic region, and (**K**) lumbar region. POWV-infected mouse with (**C**) meningoencephalitis (red arrow) in cervical region, (**F**) absent and degenerating motor neuron (black arrow) in dorsal horn, and infiltrations in (**I**) thoracic and (**L**) lumbar regions.

Powassan virus-infected brains showed meningoencephalitis (Figure 2I) that included the cervical spinal cord (Figure 4C), while the meninges of the spinal cord appeared normal in the DTV-infected samples (Figure 4B). The cervical spinal cords of the POWV-infected mice exhibited considerable histopathology in which the nuclei of ventral gray matter motoneurons were either shrunken with irregular cell bodies or had entirely degenerated (Figure 4F). There was also conspicuous extravasation of inflammatory cells in spinal cord gray matter of the POWV-infected mice, especially in the cervical spinal cord (Figure 4C,F,I,L). In contrast, the neurons in the cerebellum and spinal cord of the DTV-infected mice appeared normal in their numbers and morphology (Figure 2B and Figure 4B,E). 

**Figure 5:**
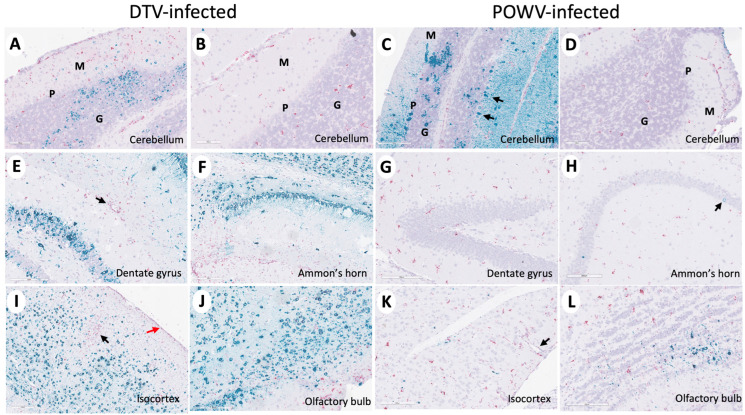
Divergent patterns of infection in DTV- and POWV-infected mouse brains. Brains were harvested at the time of euthanasia. Representative images of DTV-infected (**A**,**B**,**E**,**F**,**I**,**J**) and POWV-infected (**C**,**D**,**G**,**H**,**K**,**L**) brains stained for viral RNA (teal) and CD11b^+^ cells (pink). (**A**–**D**) Molecular (green M), Purkinje (green P), and granular (green G) cell layers of the cerebellum and (**C**) POWV-infected Purkinje cells (black arrow). (**E**) CD11b positive cells (black arrow) around a vessel in the hippocampal region of a DTV-infected mouse brain. (**H**) Minimal infected cells (black arrow) in the hippocampal region of a POWV-infected mouse brain. (**I**) CD11b positive cells around a vessel (black arrow) and in the leptomeninges (red arrow) of the isocortex of a DTV-infected mouse brain. (**K**) CD11b positive cells in the perivascular (Virchow–Robin) space (black arrow) of a POWV-infected mouse brain.

The brain sections stained for viral RNA and CD11b+ cells were examined and scored for severity in a manner consistent with histopathological analysis (Table S3). The Powassan virus and DTV infection of neurons were noted throughout all the infected mouse brains; however, the regions infected differed between the viruses. The brains of mice infected with the POWV exhibited staining of viral RNA in all regions but were concentrated primarily in the cerebellum and brainstem (Figure 5C). The Powassan virus only rarely infected the hippocampus, isocortex, and olfactory bulb in most animals (Figure 5G,H,K,L) compared to relatively high levels of infection of Purkinje cells and granule cells in in the cerebellum (Figure 5C). 

The deer tick virus-infected brains showed a markedly different pattern of viral infection. There was reduced infection in the cerebellum and brainstem (Figure 5A,B) in comparison to the POWV-infected brains but a pronounced increase in viral RNA found in the neurons in the isocortex, olfactory bulb, and hippocampal formation including the dentate gyrus granule cells and pyramidal neurons in Ammon’s horn (Figure 5E,F,I,J). The virus severity scores show increased virus in the cortex, hippocampal formation, and thalamus of DTV-infected mice and increased virus in the cerebellum of POWV-infected mice (Figure 3A,B). The cD11b severity scores show increased positive cells in the hippocampal formation of DTV- compared to POWV-infected mice (Figure 3C). 

**Figure 6:**
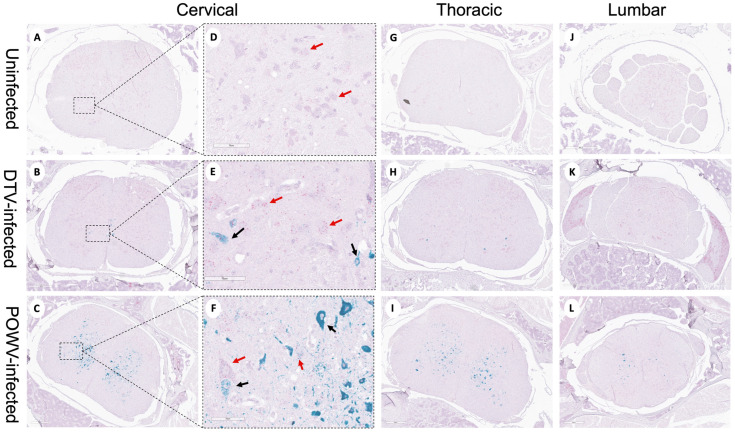
Divergent patterns of infection in DTV- and POWV-infected mouse spinal cords. Spinal cords were harvested at the time of euthanasia. Representative images of uninfected, DTV-infected, and POWV-infected mouse spinal cord with teal staining of viral RNA and pink staining of neuronal nuclei (NeuN). Panels (**D**–**F**) are enlarged images of the region marked by dotted square in panels (**A**–**C**). Uninfected cervical (**A**,**D**), thoracic (**G**), and lumbar (**J**) regions with normal motor neurons shown (**D**, red arrows). DTV-infected mouse spinal cord cervical (**B**,**E**), thoracic (**H**), and lumbar (**K**) regions with minimal infected neurons. POWV-infected mouse spinal cord cervical (**C**,**F**), thoracic (**I**), and lumbar (**L**) regions with decreasing infection across the cord and degenerating motor neurons ((**F**), black arrows).

Neuron-specific staining for virus differed in the spinal cords of POWV- and DTV-infected mice. In DTV-infected mice, minimal viral RNA was present across the cervical, thoracic, and lumbar regions (Figure 6B,E,H,K). In sharp contrast, prominent infection of the cervical spinal cord neurons of the POWV-infected mice was consistent with contiguous brainstem, cerebellum and midbrain regions that were also positive for POWV. The presence of viral RNA-containing neurons decreased considerably from the cervical to thoracic and lumbar regions in several animals indicating that the cervical spinal cord was particularly affected at this stage of infection (Figure 6C,F,I,L). One POWV-infected mouse had a prominent infection of the hippocampus and isocortex (Table S3); however, this animal also had the characteristic lower brain stem and spinal cord infection seen in the other POWV-infected mice. 

In summary, infection of the brainstem, cerebellum, and contiguous cervical spinal cord by the POWV correlates with the observed clinical signs seen during POWV infection, in which animals exhibited substantial paralysis and motor deficits while infection with the DTV was concentrated mainly to the cerebral cortex, olfactory bulbs, and midbrain with less lower brainstem and spinal cord involvement. The distinct regional patterns of infection by the DTV and POWV are depicted schematically in Figure 3B.

### 2.5. Comparative Transcriptomics of POWV and DTV 

Transcripts from either DTV- or POWV-infected brains were generated and compared against the uninfected controls and represented increased levels of inflammatory cytokine and chemokine genes (Figures S3A,B and S4). The smallest *p*-adjusted value for either group was STAT1, with genes involved in immunological signaling, inflammation, and interferon response following infection (Figure S3C,D). The 25 genes with the smallest *p*-values in POWV-infected brains are involved with interferon signaling, antigen presentation, and viral response (Figure S6C,D). There was a significant upregulation in the genes responsible for controlling viral infection in both the samples, including interferon-stimulated genes and innate immune-signaling genes associated with MyD88- and RIG-I -like receptors. Individually, both the POWV and DTV appear to activate the JAK-STAT pathway through TLR stimulation and the interferon-induced anti-viral state.

**Figure 7:**
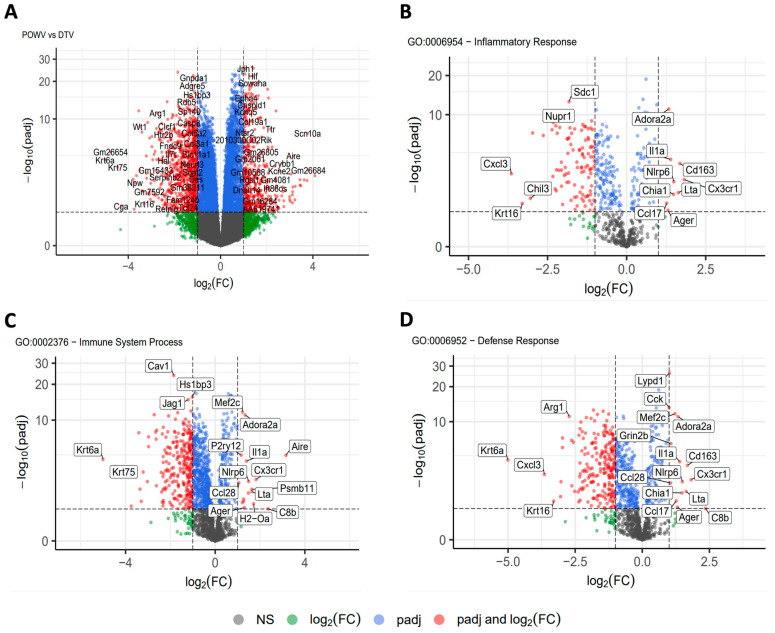
RNA sequencing analysis of brain tissue. Brains were harvested at the time of euthanasia and stored in trizol before homogenization and RNA extraction followed by RNA-Seq. Transcript libraries were analyzed using R with (**A**) POWV compared to DTV. Genes were separated into smaller gene ontology sets and compared for (**B**) inflammatory response, (**C**) immune system process, and (**D**) defense response.

The transcript libraries generated from the POWV- and DTV-infected brains were then compared (Figure 7A), and smaller libraries were generated based on GO enrichment analysis terms: inflammatory response (Figure 7B), immune system process (Figure 7C), and defense response (Figure 7D). The brain tissues infected with the POWV had significantly higher levels of inflammatory pathway genes including NLRP6 and chemokines associated with hematopoietic inflammatory cell recruitment including CX3CR1. The tissues infected with the DTV displayed higher levels of Cav1, which induces NF-κB through T-cell activation. Furthermore, there is a strong difference in the levels of CXCL3, with DTV-infected tissues having significantly higher levels of this neutrophil chemotactic cytokine. The results from this comparison suggest that while there is a similar pattern to the interferon response and establishment of the anti-viral state, DTV and POWV infections of the CNS involve the recruitment of different immune cell types and the activation of different inflammatory pathways. 

### 2.6. Cytokine and Chemokine Analysis of Terminal Serum

**Figure 8:**
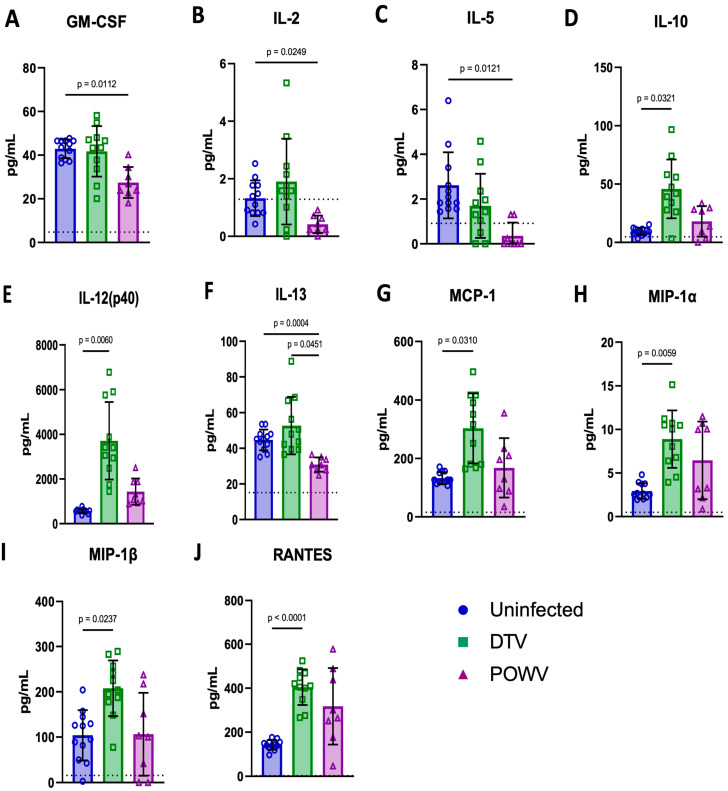
Cytokine and chemokine levels were measured in terminally collected serum from uninfected (*n* = 6M/6F), DTV-infected (*n* = 5M/6F), and POWV-infected (*n* = 3M/5F) mice by Bio-Plex Mouse Cytokine 23-plex panel. Significant differences were present between uninfected and POWV-infected mice for four parameters (**A**,**B**,**C**,**F**), between uninfected and DTV-infected mice for six parameters (**D**,**E**,**G**,**H**,**I**,**J**), and between DTV- and POWV-infected mice for one parameter (**F**). Comparisons were made using Brown–Forsythe and Welch’s ANOVA test with Dunnett’s T3 multiple comparisons test. The horizontal dotted lines in the individual graphs represents the limit of detection for each of the cytokines and chemokines.

The levels of 23 analytes were measured in terminal blood samples to determine the differences between the uninfected (*n* = 12), DTV-infected (*n* = 11), and POWV-infected (*n* = 8) mice (Figure 8 and Figure S2). There were significantly higher levels of six analytes in the DTV-infected groups compared to the uninfected groups: IL-10 [*p* = 0.0321] (Figure 8D), IL-12(p40) [*p* = 0.006] (Figure 8E), MCP-1 [*p* = 0.031] (Figure 8G), MIP-1α [*p* = 0.0059] (Figure 8H), MIP-1β [*p* = 0.0237] (Figure 8I), and RANTES [*p* = <0.0001] (Figure 8J). In contrast, there were only significantly lower levels in four analytes in the POWV-infected animals compared to the controls: GM-CSF [*p* = 0.0112] (Figure 8A), IL-2 [*p* = 0.0249] (Figure 8B), IL-5 [*p* = 0.0121] (Figure 8C), and IL-13 [*p* = 0.0004] (Figure 8F). The only difference between the DTV and POWV was in IL-13 [*p* = 0.0451] (Figure 8F). These data indicate an entirely different proinflammatory response in the periphery following infection by these two viruses. The analytes with no significant differences are shown in Figure S2.

## 3. Discussion

Flaviviruses are endemic across much of the globe and cause millions of cases of human illness each year [22]. The severity of neurotropic flaviviruses such as the West Nile virus (WNV), Japanese encephalitis virus (JEV), TBEV, and Powassan virus can range from mild to fatal and can cause severe neuroinvasive disease with long-term sequelae in a high percentage of survivors. For example, the disease severity, fatality rate, and long-term sequelae associated with TBEV infection has been shown to vary depending on the subtype despite the high sequence similarity between these viruses [23,24]. Similarly, the DTV and POWV share approximately 94% of amino acid identity and 84% of nucleotide sequence similarity and are serologically indistinguishable [6,7,8]. Coupled with the lack of a vaccine or approved treatment and overlapping circulation in the United States, testing to differentiate lineages is typically not performed for human cases. 

### 3.1. Immunocompetent Mice Inoculated with Powassan Virus and Deer Tick Virus Have Different Clinical Disease Presentations

In this report, we have shown that the DTV and POWV do not have the same clinical presentation when inoculated via the same route and dosage. Common clinical signs in both the virus groups included ruffled fur, hunched posture, ocular discharge, and weight loss. The degree of weight loss experienced by the DTV-infected mice was more severe than that of the POWV-infected mice. Seizure was the most common neurological symptom present in the DTV-infected mice and was consistent with prominent infection and inflammation in the hippocampus. The mice infected with the POWV experienced neurological signs more consistent with infection and inflammation of the lower brainstem, cerebellum, and spinal cord including paresis and paralysis. 

### 3.2. Powassan Virus and Deer Tick Virus Induce Neuronal Death in Differing Regions of the Brain 

The reduced levels of CD11b^+^ cells seen in the histopathology of the POWV-infected brains as compared to the DTV-infected brains are congruent with the histopathology and spatial distribution of the virus. The spatial distribution of the POWV in the brain is concentrated in the lower brain and spinal cord, while the DTV preferentially infected and induced inflammation in the hippocampus and isocortices, which suggests a different neuroinvasive route and/or spread of infection in the brain. The neurologic clinical signs exhibited during late-stage infections are dissimilar as well, with the DTV eliciting seizures, while POWV neuropathology is manifested as motor deficits associated with paralysis. The comparative neuropathology of the POWV and DTV reveals a stark contrast which is recapitulated in chemokines, histopathology, and observed clinical signs; the DTV infects the hippocampus and isocortex, while POWV infection is concentrated within the cerebellum, brain stem, and spinal cord. Interestingly, acute seizures have been linked to the loss of the pyramidal cell layer and neuronal loss in the hippocampus in Theiler’s murine encephalomyelitis virus-infected mice, which is consistent with pathology findings for DTV-infected mice that experienced seizures in the present work [25,26]. Published human cases of fatal DTV infection have shown severe inflammation and infection of the cerebellum and brainstem which is more consistent with neuropathology present in POWV-infected mice [14,15]. In these human cases, limb weakness and difficulty ambulating were noted and are consistent with the deficits we observed with the mice with heavy infection in the cerebellum, brainstem, and cervical spinal cord. 

The changes in the clinical presentation of disease and pattern of infection in the CNS could be attributed to a variety of factors, including person-to-person variability, the site of infection, and the genomic diversity of viruses. Other flaviviruses have demonstrated that genetic diversity in the E and NS5 regions change the phenotype of the virus including immunogenicity and pathogenicity [27]. The strain of the POWV or DTV used for inoculation, route and method of inoculation, and time replicating in the periphery could affect the clinical signs and response to infection [21,23,28,29,30]. This is underscored by our findings in that the vectored route of inoculation can cause a diverse set of clinical signs but ultimately be relegated to either a cortical region meningoencephalitis or involve the cerebellum and spinal cord, resulting in descending myelitis.

### 3.3. Host Chemokine and Inflammatory Response to Powassan Virus Is Different Compared to Deer Tick Virus

The neuroinvasion of flaviviruses has been shown to be a result of inflammatory breakdown of the blood–brain barrier; however, other mechanisms may result in CNS infection as well. Our findings are consistent with previous studies showing an upregulation of STAT1 and genes associated with inflammation and interferon in the brain of moribund animals. The histopathological differences seen between the regions of the brain infected by the DTV and POWV suggest a spatial tropism which may be affected by a region-specific neuroinvasion and dissemination. The olfactory bulb of DTV-infected animals had high levels of virus present which suggests that this area was significant for viral replication and consequent spreading to cerebral cortex and to the underlying midbrain structures. The clinical signs in POWV-infected animals combined with the histopathology clearly show myelitis and destruction of neurons in the ventral horns of the cervical spinal cord, contiguous lower brain stem, and cerebellum, illustrating a differing spatial tropism compared to the DTV. The differences in these spatial tropisms could be explained by differential routes of neuroinvasion, with DTV for instance preferentially crossing the cribiform plate from the olfactory neurons to infect the olfactory bulb, while the POWV may utilize retrograde axonal transport of the virus from the periphery into the spinal cord by the neural route or perhaps by a unique viremic route initiated by preferential inflammatory degradation of the BBB in the spinal cord. 

The comparative RNA-Seq data suggest that there are different innate immune pathways responding to the DTV and POWV during CNS infection. While both these viruses elicit a strong interferon response, there is a significant increase in NLRP6 and IL1α transcripts in the tissues infected with the POWV relative to tissues infected with the DTV. There is also a significant increase in CX3CR1 and AIRE genes which indicate the recruitment of CD8+ T-cells in POWV-infected tissues. The DTV-infected tissues conversely exhibit significant increases in CXCL3, CD177, and CAV1, suggesting neutrophilic recruitment combined with T-cell dependent NF-κB transcription. The spatial tropism exhibited in the histopathology is supported by the levels of Krt6a seen in DTV-infected tissues, which exhibit anti-microbial properties in the olfactory bulb, an area with extensive DTV infection. These findings suggest that the pathology seen in the POWV is driven by CD8+ cytotoxic T-lymphocytes, while the DTV elicits repetitive NF-κB-mediated transcription and inflammation. These findings are congruent with recent studies that suggest that the POWV is less inflammatory in neuronal cell cultures and that the pathogenesis of the POWV, like the TBEV, is driven by the extravasation and activity of cytotoxic T-lymphocytes [20,31]. The response to the POWV has recently been expanded with experiments examining the role of TRIM5 in POWV infection [32]. We have also noted in this report that TRIM5 is upregulated in both DTV and POWV infections compared to the uninfected controls but that this increase is significantly higher in response to the POWV. This comparative view of RNA transcriptomics in DTV and POWV infections illustrate a difference in cellular and inflammatory responses to infection. As a potential limitation of this study, these conclusions are based on observations of tissues harvested in moribund mice or when mice met euthanasia criteria. This has resulted in comparisons of histopathological and RNA-Seq data based on the ultimate pathological outcomes. Comparing the tissue data on specific days post-infection might provide additional information on POWV and DTV infections in the CNS, which will be determined in future studies.

In conclusion, a direct comparison of the POWV (lineage I) and DTV (lineage II) demonstrated distinct differences in their clinical presentation and neuro-immunopathology. These differences suggest these two viral lineages do not utilize the same routes of neuroinvasion and stimulate distinct neuroimmunological responses. 

## 4. Methods

### 4.1. Ethics Statement

Experiments on mice were conducted in an American Association for the Accreditation of Laboratory Animal Care (AAALAC)-approved facility within the Animal Biosafety Level 3 (ABSL3) containment. All procedures were performed in accordance with SUNY Upstate Medical University Institutional Animal Care and Use Committee approved protocol (#455) which adheres to the Guide for the Care and Use of Laboratory Animals [33].

### 4.2. Viruses, Animals, and Infection

Deer tick virus (Spooner strain) and POWV (LB strain) stocks were provided by the World Reference Center for Emerging Viruses and Arboviruses at University of Texas Medical Branch and had been previously passaged once in suckling mouse brains. The stocks were then passaged six times on Vero E6 cells. The Vero E6 cells (CRL-1586; American Type Culture Collection) were cultured using Modified Eagle’s Medium (MEM; Corning) and supplemented with a fetal bovine serum (FBS), non-essential amino acids, and a penicillin/streptomycin antibiotic mixture and maintained at 37 °C with 5% CO_2_. Virus titers were determined by a focus-forming assay as previously described [34].

Next-generation sequencing demonstrated that the consensus nucleotide sequence of our DTV stock was 99.84% identical to the DTV Spooner, Wisconsin isolate (GenBank: HM440560.1), and our POWV stock was 99.96% identical to the POWV LB, Ontario isolate (GenBank: NC_003687.1). Of the 17 nucleotide differences between our DTV stock and DTV Spooner isolate, there were 3 resulting amino acid sequence differences in M (N213K), NS4B (S2368T), and NS5 (K2903R). There were three nucleotide differences between our POWV stock and POWV LB isolate resulting in one amino acid sequence difference in E (Q1436R) as well as one nucleotide gap which may have resulted in one amino acid sequence difference in NS5 (W8255 to L or S).

Thirty-six 5-week-old male (*n* = 18) and female (*n* = 18) BALB/cJ mice were purchased from The Jackson Laboratory (Bar Harbor, ME). Upon receipt, the mice were housed three per cage in individually ventilated cages with HEPA-filtered supplies and exhaust air. Food and water were provided *ad libitum*. The animals were provided a 12 h light/12 h dark cycle within a temperature- and humidity-controlled room. The mice were acclimated to the facilities for one week. On the day of study initiation, prior to infection, the animals were observed and weighed to ensure there were no apparent physical abnormalities and that each group had a similar weight distribution. The uninfected control mice had an average weight of 21.43 g (±2.09), the DTV-infected mice had an average weight of 20.74 g (±1.98), and the POWV-infected mice had an average weight of 21.06 g (±2.16). 

All the mice received a single footpad injection of 30 µL under isoflurane anesthesia. The control mice received serum-free Dulbecco’s Modified Eagle Medium (DMEM, Corning), and the infected mice received 10^3^ focus-forming units (FFU) of either POWV Lineage I (POWV LB) or Lineage II (DTV Spooner). Each group contained six male and six female mice. Bodyweight measurements and detailed clinical observations were performed daily to assess the animals’ appearance, behavior, respiration, and neurological signs of disease (Table S1). Each mouse was assigned a cumulative clinical score based on the scoring system shown in Table S1. Submandibular blood collection was performed from Day 0 to 7 using alternating cohorts of mice and alternating sites. Terminal blood was collected via cardiac puncture following euthanasia. 

When the animals reached the criteria for humane euthanasia or at 11 days post-infection (dpi), euthanasia was performed by CO_2_ inhalation followed by cervical dislocation. Whole-body perfusion was performed using 10 mL of sterile 0.9% sodium chloride, USP. The tissues collected at necropsy were stored in either Buffer RLT (Qiagen, Germantown, MD, USA) or 10% Neutral Buffered Formalin (NBF). The blood was stored in Buffer RLT. The samples stored in Buffer RLT were maintained in ABSL3 facilities for a minimum of 24 h to allow for inactivation. The samples stored in 10% NBF were fixed for a minimum of 72 h, with one change in NBF occurring 24 h after collection. 

No specific methods were used to minimize confounders or blind staff to group allocation during the experiment. Each day, the procedures were performed with the uninfected animals handled first, followed by the DTV-infected animals, and the POWV-infected animals were handled last. Equipment and PPE were cleaned or changed between the infected groups to prevent cross-contamination.

### 4.3. Viral RNA Detection via RT-qPCR

Total RNA was extracted from the blood and tissue samples using RNeasy Mini Kits (Qiagen) using previously established modifications to the kit protocol [35]. The RNA was quantified using a DS-11+ Spectrophotometer (Denovix, Wilmington, NC, USA) and tested for the presence of viral RNA by RT-qPCR on a Bio-Rad CFX96 Touch Real-Time PCR System. Viral RNA was detected with forward (CCGAGCCARRGTGAGGATGT), reverse (TCTTTTGCYGARCTCCACTT), and probe (TTCATAGCGAAGGTKAGRTCCAACG) primers which have been previously validated to detect a region of the NS5 gene conserved between the POWV and DTV [36]. Standard curves for the POWV and DTV were generated from 10-fold dilutions of a known quantity of viral RNA and used to estimate the viral burden in experimental samples. 

### 4.4. Histology and RNA In Situ Hybridization

Upon reaching humane euthanasia criteria (Figure 1D and Table S1), the tissues were harvested and used for the procedures described below. The inactivated tissues from three male and three female mice per group were transferred to 70% ethanol and shipped to Histowiz, Inc. (Brooklyn, NY, USA). for embedding and sectioning. The brains were sectioned at 5 µM thicknesses and mounted on glass slides. Hematoxylin and eosin staining was performed at Histowiz, Inc in accordance with their established methods. RNA in situ hybridization was performed using RNAscope 2.5 HD Duplex kits (catalog 322430, Advanced Cell Diagnostics, Newark, NJ, USA) per manufacturer recommendations. The brain sections underwent dual-staining protocol for POWV-positive-sense RNA probe (catalog 415641, Advanced Cell Diagnostics, Newark, NJ, USA) and *Mus musculus* integrin alpha M/CD11b (catalog 311491, Advanced Cell Diagnostics, Newark, NJ, USA) to identify infected cells and innate leukocytes. The spine sections underwent the dual-staining protocol using the same viral RNA probe used on the brain sections and *Mus musculus* RNA-binding protein, fox-1 homolog (*C. elegans*) 3/NeuN (catalog 481707, Advanced Cell Diagnostics, Newark, USA), to identify infected cells including NeuN^+^ neurons. All the slides were counterstained with a 50% solution of Gill’s hematoxylin I. The positive control samples were processed using *Mus musculus* Duplex Ppib and Polr2a (catalog 321651, Advanced Cell Diagnostics, Newark, NJ, USA) for process verification. Both Hematoxylin and eosin staining and RNA in situ hybridization were performed on all the brains sectioned.

The sagittal brain sections were examined in a rostro-caudal direction, including the olfactory bulb, cerebral cortex, hippocampus, thalamus, hypothalamus, midbrain, pons, medulla oblongata, and Purkinje, granular, and molecular layers of the cerebellum. Microscopic lesions, including microgliosis, perivascular cuffing, and neuronal degeneration were graded as 0 (absence of lesions); 1 (minimal); 2 (mild); and 3 (marked). The presence of meningitis, encephalitis, and meningoencephalitis were noted as M, E, and ME, respectively. RNAscope-stained cells were graded a 0 (absence of staining); 1 (very few to low); 2 (moderate); and 3 (numerous). Areas that were not present were noted as NP.

### 4.5. RNA Sequencing and Bioinformatics

RNA sequencing and bioinformatics were performed for the same three male and three female mice per group which had H&E and RNA in situ hybridization performed. The total RNA was quality checked using an Agilent Fragment Analyzer to assess RNA quality and Qubit Fluorescence (Invitrogen, Carlsbad, CA, USA) to measure its concentration. RNA libraries (200ng total starting material) were prepared following the Illumina RiboZero Total Stranded RNA library prep kit (Illumina, San Diego, CA, USA)using the ribosomal removal protocol. Following library preparation, concentration, and quality control, the final libraries were pooled to 10 nM, and the concentration of the pool was determined using the QuantaBio Universal qPCR reaction kit (QuantaBio, Beverly, CA, USA). After dilution and denaturing, the pooled library was loaded onto an Illumina NovaSeq6000 (PE100) for sequencing at 250 pM as the final concentration. 

Per-cycle base call (BCL) files generated by the Illumina NovaSeq were converted to per-read FASTQ files using bcl2fastq version 2.20.0.422 using the default parameters. The quality of the sequencing was reviewed using FastQC version 0.11.6. Detection of potential contamination was performed using FastQ Screen version 0.14.1. Finally, FastQC and FastQ Screen quality reports were summarized using MultiQC version 1.9.0. 

Adapter trimming was not performed. Genomic alignments were performed using HISAT2 version 2.2.1 using default parameters. Ensembl reference GRCm38 was used for the reference genome and gene annotation set. Sequence alignments were compressed and sorted into binary alignment map (BAM) files using sam tools version 1.3. Counting of mapped reads for genomic features was performed using Subread feature Counts version 2.0.0 using the parameters-p-s 2 –g gene_name –t exon –Q 60-B-C, the annotation file specified with –a was the Ensembl GRCm38 reference provided by Illumina’s iGenomes. Alignment and feature assignment statistics were summarized using MultiQC. 

Differentially expressed genes were detected using the Bioconductor package DESeq2 version 1.38.1. The significance was set to an adjusted *p*-value less than or equal to 0.05. DESeq2 tests were run for differential expression using a negative binomial generalized linear models, dispersion estimates, and logarithmic fold changes. DESeq2 calculated log2 fold changes and Wald test *p*-values as well as performed independent filtering and adjusted for multiple testing using the Benjamini–Hochberg procedure to control the false discovery rate (FDR).

A gene ontology analysis was performed using the GOSeq Bioconductor package version 1.50.0. GOSeq performs a gene ontology analysis while addressing biases present in RNA-Seq data not found using other techniques, namely, that the expected read counts for a transcript are based on both the gene level of expression and the length of the transcript. While other tools are commonly used for GO term analysis, they do not account for this effect leading to results that are biased toward GO terms with longer transcripts. The Wallenius approximation used by GOSeq approximate the GO term enrichment and calculated *p*-values for each GO category being overrepresented among genes that were differentially expressed. These *p*-values were corrected for multiple testing using the Benjamini–Hochberg procedure.

### 4.6. Cytokine and Chemokine Analysis of Terminal Serum

Terminal serum samples were tested in duplicate for cytokine and chemokine levels on a Bio-plex 200 (Bio-Rad) using a Bio-plex Pro Mouse Cytokine 23-plex assay according to kit instructions. The cytokines detected were IL-1α, IL-1β, IL-2, IL-3, IL-4, IL-5, IL-6, IL-9, IL-10, IL-12 (p40), IL-12 (p70), IL-13, IL-17A, eotaxin, G-CSF, GM-CSF, IFN-γ, KC, MCP-1 (MCAF), MIP-1α, MIP-1β, RANTES, and TNF-α. The serum was diluted 1:4 in the kit supplied diluent, incubated with cytokine-specific labeled beads and detected with biotinylated antibody and streptavidin-PE. 

### 4.7. Statistical Analysis

Statistical analysis was conducted with GraphPad Prism software version 9.4.1 (GraphPad). No data were excluded from the analysis.

## Data Availability

All relevant data are within the manuscript and its Supporting/Supplemental Files.

## Supplementary Materials

The following supporting information can be downloaded at: https://www.mdpi.com/article/10.3390/v16060820/s1, Figure S1. Comparison of differences between male and female mice infected with DTV or POWV. Male (*n* = 6) and female (*n* = 6) DTV-infected mice had significantly different clinical observation scores (A) 7 dpi at the AM observation [*p* = 0.0298] and changes to body weights (B) 3 dpi [*p* = 0.04673]. No significant differences were found in survival (C), or viral RNA detected in tissues (D) or blood (E). Male (*n* = 6) and female (*n* = 6) POWV-infected mice had significantly different changes to body weights (G) at 4 dpi [*p* = 0.03196] and differences in the amount of viral RNA present in their livers (I) [*p* = 0.0257]. No significant differences were found in the clinical observation scores (F), survival (H), or viral RNA present in the blood (J). Comparisons were made using unpaired, two-tailed Student’s *t*-test with Welch’s correction. Error bars show SEM. Figure S2. Cytokine and chemokine levels were measured in terminally collected serum from uninfected (*n* = 6M/6F), DTV-infected (*n* = 5M/6F), and POWV-infected (*n* = 3M/5F) mice by Bio-Plex Mouse Cytokine 23-plex panel. No significant differences were found between groups for 13 analytes. Comparisons were made using Brown–Forsythe and Welch’s ANOVA test with Dunnett’s T3 multiple comparisons test. Figure S3. RNA sequencing analysis of brain tissue. Brains were harvested at moribundity and stored in trizol before homogenization and RNA extraction followed by RNA-Seq. Transcript libraries were analyzed using R with (A) POWV and (B) DTV compared to uninfected controls and the 25 smallest adjusted *p*-values represented in a heat map for (C) POWV and (D) DTV. Figure S4. GO Term analysis of RNA sequencing from infected brain tissue. Transcripts from previous RNA sequencing (Figure 4 and Figure 6) analyzed using Kyoto Encyclopedia of Genes and Genomes (KEGG) using R for the top 25 overrepresented GO terms for (A) POWV and (B) DTV compared to uninfected controls and (C) POWV compared to DTV. Table S1. Clinical observation categories and scores. The highest scoring observation within a category determined the score for the category. Scores from each category were added to determine the total score. Table S2. Distribution and severity of histologic lesions in brains. H&E-stained brain sections were assessed for microscopic lesions including microgliosis (MG), perivascular cuffing (PC), and neuronal necrosis (NN). Semi-quantitative scoring is indicated by 0 (absence of lesions), 1 (very few to low), 2 (moderate), and 3 (numerous). Meningitis (M), encephalitis (E), and meningoencephalitis (ME) were also noted. Areas that were not present were noted as NP. Table S3. Presence of viral RNA and CD11b in the brain via RNAscope. Staining for viral RNA (teal) and CD11b (pink) was performed using RNAscope. Brain regions were semi-quantitatively scored with 0 (absence of staining), 1 (very few to low), 2 (moderate), and 3 (numerous). Meningitis (M), encephalitis (E), and meningoencephalitis (ME) were also noted. Areas that were not present were noted as NP. Table S4. RNA-Seq full list of comparisons by gene for POWV vs. DTV group. Table S5. RNA-Seq full list of comparisons by gene for POWV vs. control group. Table S6. RNA-Seq full list of comparisons by gene for DTV vs. control group.
