# Supplementary material for: Comparative Pathogenesis of Two Lineages of Powassan Virus Reveals Distinct Clinical Outcome, Neuropathology, and Inflammation
Source: Viruses. 2024 May 22;16(6):820. doi: 10.3390/v16060820 (PMC11209061; doi:10.3390/v16060820)
Supplement: Supplementary file 1 [file viruses-16-00820-s001.zip › Table S2 - H&E lesion scoring.pdf]

Table S2. Distribution and severity of histologic lesions in brains.

Group	Animal	Sex	Olfactory Bulb	Cerebral Cortex	Hippocampal Formation	Thanalmus	Hypothalamus	Midbrain	Pons	Medulla	Cerebellum			Meningitis/ encephalitis
											Purkinje cells	Granule cells	Molecular cells	
Uninfected	1001	M	-	-	-	-	-	-	-	-	-	-	-	
	1002	M	-	-	-	-	-	-	-	-	-	-	-	
	1003	M	-	-	-	-	-	-	-	-	-	-	-	
	1007	F	NP	-	-	-	-	-	-	-	-	-	-	
	1008	F												
	1009	F	-	-	-	-	-	-	-	-	-	-	-	
DTV	2001	M	+ MG, NN	++ MG, PC, NN	++ MG, PC, NN	++ MG, PC	+ MG	++ MG, PC	NP	NP	-	+ NN	+ MG	ME
	2002	M	+ MG, NN	++ MG, PC, NN	+ MG, PC, NN	++ MG, PC	+ MG, NN	++ MG	NP	NP	NP	NP	NP	ME
	2003	M	-	++ MG, PC, NN	+ MG, PC, NN	++ MG, PC, NN	++ MG, PC, NN	++ MG, PC, NN	+ MG, PC	++ MG, PC	+ NN	+ NN	++ MG, PC	ME
	2007	F	++ MG, NN	+++ MG, PC, NN	+++ MG, PC, NN	++ MG, PC, NN	++ MG, PC, NN	++ MG, PC, NN	+ MG, PC, NN	NP	-	+ NN	+ MG	ME
	2008	F	+ MG, NN	+++ MG, PC, NN	++ MG, PC, NN	++ MG, PC, NN	++ MG, PC, NN	++ MG, PC	+ MG, NN	+ MG, PC	-	-	+ MG	ME
	2009	F	+ MG	+ MG, PC, NN	+ MG, PC, NN	+ MG, PC	+ MG PC	+ MG, PC	NP	NP	-	-	+ MG, PC	ME
POWV	3001	M	NP	+ MG, PC	-	+ MG	+ MG, PC	+ MG, PC	+ MG, PC	++ MG, PC	+ NN	+ NN	++ MG	ME
	3002	M	+ MG	+ MG	-	+ MG, PC	+ MG, PC	+ MG, PC, NN	+ MG, PC	++ MG, PC, NN	-	++ NN	+ MG	ME
	3003	M	NP	+ MG, PC, NN	-	+ MG, PC, NN	+ MG, PC	+ MG, PC, NN	+ MG	+ MG, NN	-	+ NN	+ MG	ME
	3007	F	+ MG, NN	+ MG, PC, NN	-	-	+ MG	+ MG	+ PC	+ MG, PC	-	+ NN	+ MG	ME
	3008	F	+ MG, NN	++ MG, PC, NN	++ MG, PC, NN	++ MG, PC, NN	++ MG, PC, NN	++ MG, PC, NN	+ MG, PC	++ MG, NN	-	+ NN	+ MG	ME
	3009	F	-	++ MG,	+ MG, PC	+ MG	+ MG, PC	+ MG	+ MG	+ MG, PC	-	-	+ MG, PC	ME

Table S2. Distribution and severity of histologic lesions in brains. H&E-stained brain sections were assessed for microscopic lesions including microgliosis (MG), perivascular cuffing (PC), and neuronal necrosis (NN). Semi-quantitative scoring is indicated by 0 (absence of lesions), 1 (very few to low), 2 (moderate), and 3 (numerous). Meningitis (M), encephalitis (E), and meningoencephalitis (ME) were also noted. Areas that were not present were noted as NP.
