# Supplementary material for: Comparative Pathogenesis of Two Lineages of Powassan Virus Reveals Distinct Clinical Outcome, Neuropathology, and Inflammation
Source: Viruses. 2024 May 22;16(6):820. doi: 10.3390/v16060820 (PMC11209061; doi:10.3390/v16060820)
Supplement: Supplementary file 1 [file viruses-16-00820-s001.zip › Table S3 - RNA + CD11b Scoring.pdf]

Table S3. Presence of viral RNA and CD11b in the brain via RNAscope

Group	Animal	Sex	Olfactory Bulb	Cerebral Cortex	Hippocampal Formation	Thanalmus	Hypothalamus	Midbrain	Pons	Medulla	Cerebellum			Meningitis/ encephalitis
											Purkinje cells	Granule cells	Molecular cells	
Uninfected	1001	M	-<br>+	-<br>+	-<br>+	-<br>+	-<br>+	-<br>+	-<br>+	-<br>+	-<br>-	-<br>+	-<br>+	
	1002	M	-<br>+	-<br>+	-<br>+	-<br>+	-<br>+	-<br>+	-<br>+	-<br>+	-<br>-	-<br>+	-<br>+	
	1003	M	-<br>+	-<br>+	-<br>+	-<br>+	-<br>+	-<br>+	-<br>+	-<br>+	-<br>-	-<br>+	-<br>+	
	1007	F	-<br>+	-<br>+	-<br>+	-<br>+	-<br>+	-<br>+	-<br>+	-<br>+	-<br>-	-<br>+	-<br>+	
	1008	F	NP	-<br>+	-<br>+	-<br>+	-<br>+	-<br>+	-<br>+	-<br>+	-<br>-	-<br>+	-<br>+	
	1009	F	-<br>+	-<br>+	-<br>+	-<br>+	-<br>+	-<br>+	-<br>+	-<br>+	-<br>-	-<br>+	-<br>+	
DTV	2001	M	+++<br>++	+++<br>++	+++<br>+++	+++<br>++	++<br>++	++<br>++	NP	NP	+<br>-	++<br>++	+<br>++	ME
	2002	M	++<br>++	+++<br>++	+++<br>++	+++<br>++	++<br>++	++<br>++	NP	NP	-<br>-	++<br>++	+<br>++	ME
	2003	M	+<br>++	+++<br>+++	++<br>++	++<br>++	++<br>++	+<br>++	++<br>++	+<br>++	+<br>-	+<br>++	++<br>++	ME
	2007	F	++<br>++	+++<br>++	+++<br>+++	+++<br>++	+++<br>++	+++<br>++	+++<br>++	NP	-<br>-	+<br>++	+<br>++	ME
	2008	F	+<br>++	+++<br>++	+<br>++	+++<br>++	+<br>++	++<br>++	+<br>++	+<br>++	-<br>-	+<br>++	+<br>++	ME
	2009	F	+<br>++	++<br>++	+++<br>+++	++<br>++	+<br>++	+<br>++	NP	NP	-<br>-	+<br>++	+<br>++	ME
POWV	3001	M	NP	+<br>+	-<br>+	+<br>+	+<br>++	+<br>++	+<br>++	+<br>++	+<br>-	+<br>+	++<br>++	ME
	3002	M	++<br>++	++<br>++	-<br>++	++<br>++	++<br>++	+++<br>++	++<br>++	+++<br>++	+++<br>-	+++<br>++	+++<br>++	ME
	3003	M	NP	++<br>++	-<br>++	+<br>++	+<br>++	++<br>++	+<br>++	++<br>++	+<br>-	++<br>++	+<br>++	ME
	3007	F	+<br>++	+<br>++	+<br>++	+<br>+	+<br>++	++<br>++	+<br>++	++<br>++	++<br>-	++<br>++	++<br>++	ME
	3008	F	++<br>+	+++<br>++	+++<br>++	+++<br>++	++<br>+	++<br>++	++<br>++	+++<br>++	+<br>-	++<br>++	++<br>++	ME
	3009	F	-<br>+	+<br>+++	-<br>++	+<br>++	-<br>++	-<br>++	-<br>++	+<br>++	+<br>-	+<br>++	+<br>++	ME

Table S3. Presence of viral RNA and CD11b in the brain via RNAscope. Staining for viral RNA (teal) and CD11b (pink) was performed using RNAscope. Brain regions were semi-quantitatively scored with 0 (absence of staining), 1 (very few to low), 2 (moderate), and 3 (numerous). Meningitis (M), encephalitis (E), and meningoencephalitis (ME) were also noted. Areas that were not present were noted as NP.
