# Supplementary figures and images for: Comparative Pathogenesis of Two Lineages of Powassan Virus Reveals Distinct Clinical Outcome, Neuropathology, and Inflammation
Source: Viruses. 2024 May 22;16(6):820. doi: 10.3390/v16060820 (PMC11209061; doi:10.3390/v16060820)

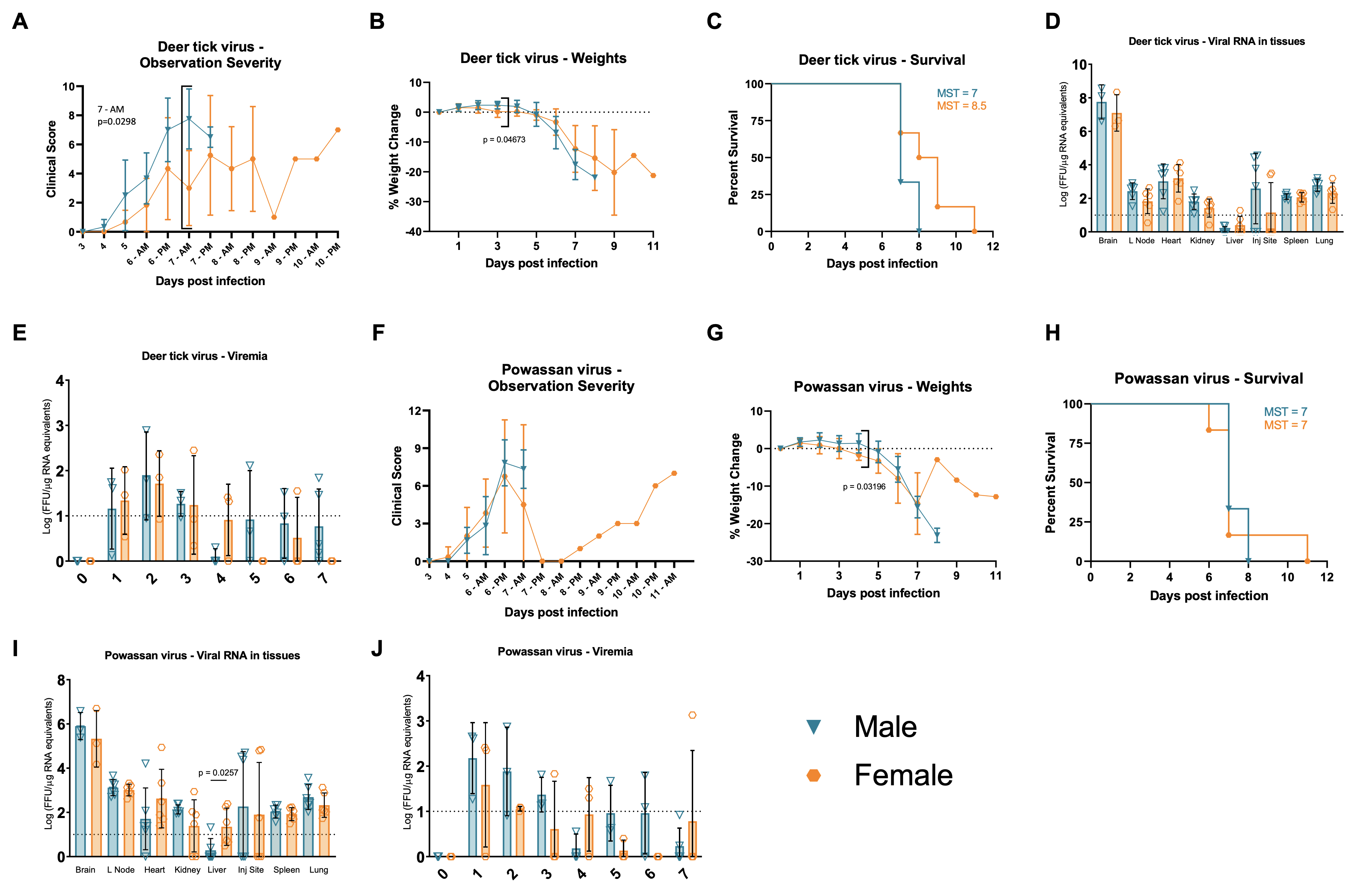

Supplement: Supplementary file 1 [file viruses-16-00820-s001.zip › Figure S1 (Male vs Female).png]

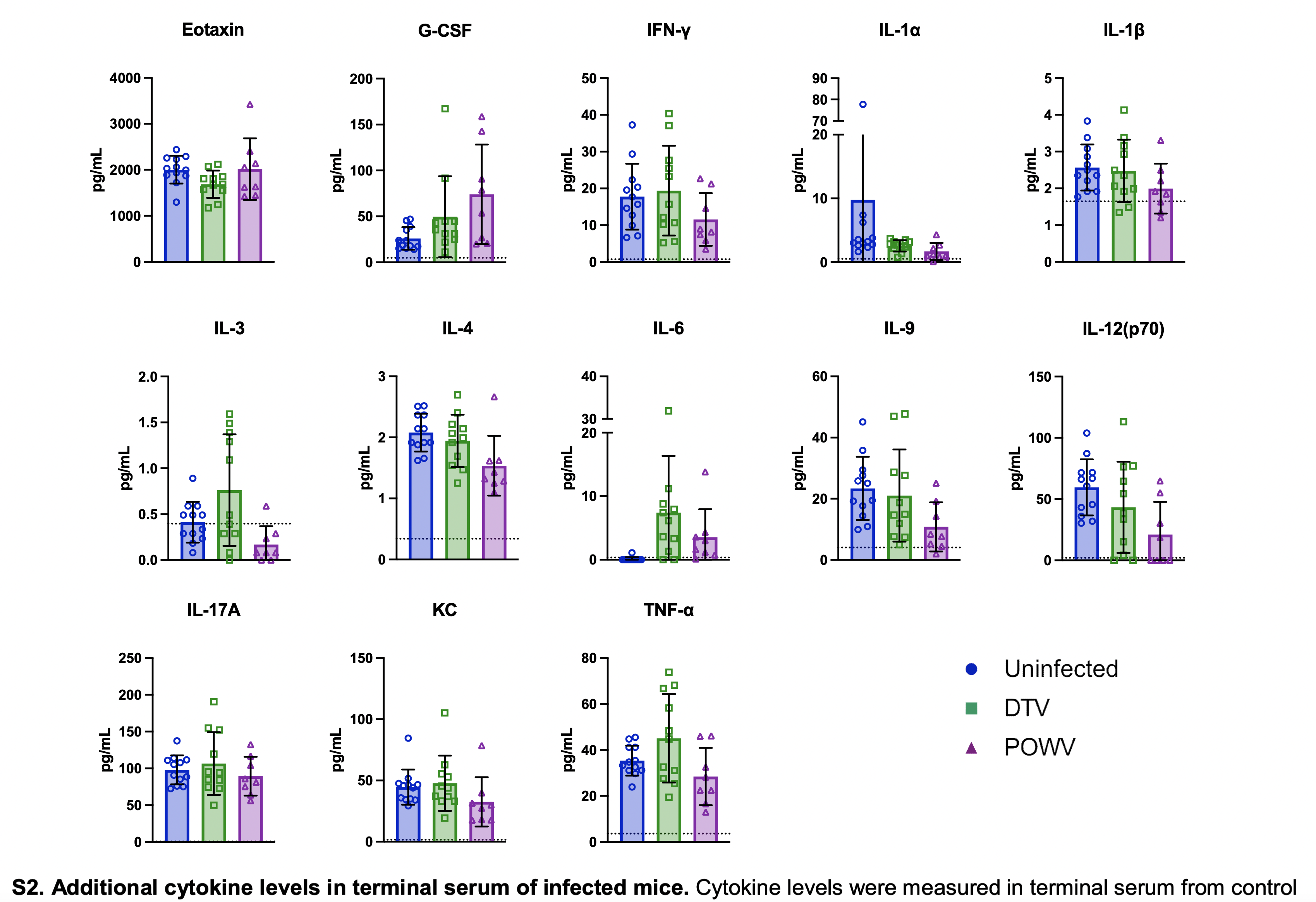

Supplement: Supplementary file 1 [file viruses-16-00820-s001.zip › Figure S2. - Bioplex.png]

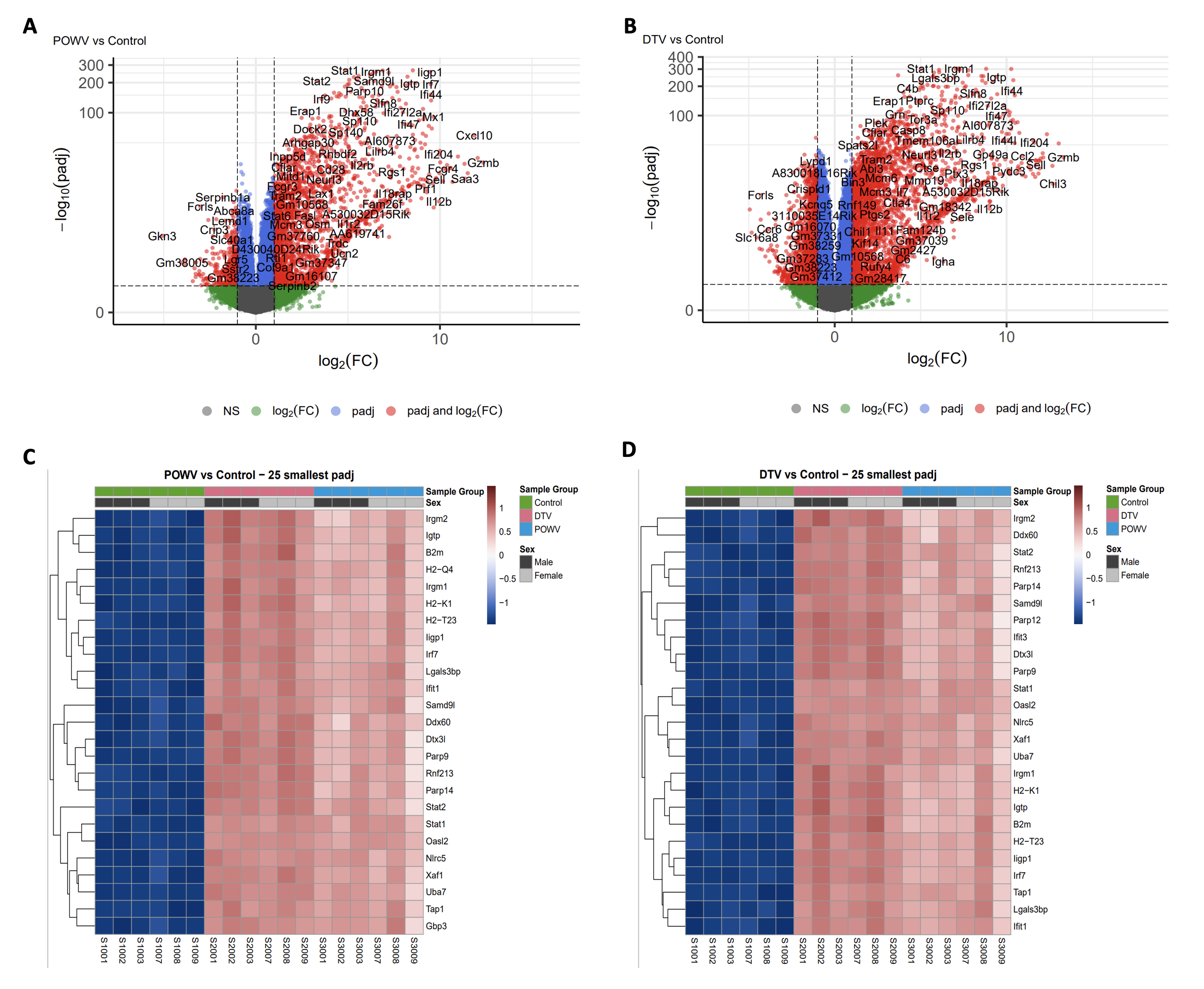

Supplement: Supplementary file 1 [file viruses-16-00820-s001.zip › Figure S3 (RNAseq).png]

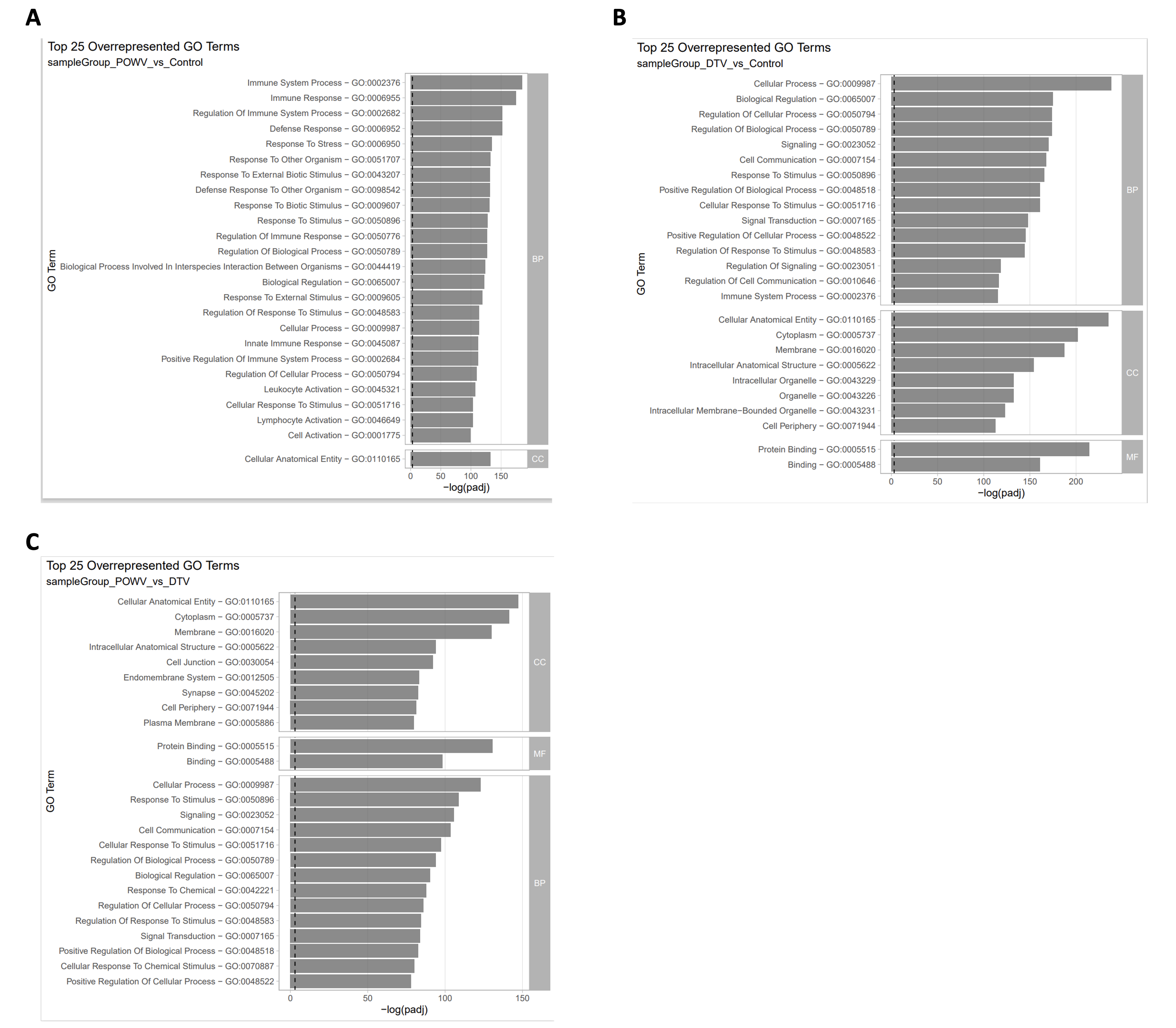

Supplement: Supplementary file 1 [file viruses-16-00820-s001.zip › Figure S4 Gene Ontology 25 terms.png]
